# Past social support influences pre-performance self-confidence and performance quality among student musicians

**DOI:** 10.3389/fpsyg.2025.1544059

**Published:** 2025-05-30

**Authors:** Michiko Yoshie, Yuki Morijiri

**Affiliations:** ^1^Department of Information Technology and Human Factors, National Institute of Advanced Industrial Science and Technology (AIST), Tsukuba, Japan; ^2^Department of Human and Engineered Environmental Studies, Graduate School of Frontier Sciences, The University of Tokyo, Kashiwa, Japan; ^3^Graduate School of Education, Tokyo Gakugei University, Tokyo, Japan

**Keywords:** music performance anxiety, stage fright, social support, music education, musician

## Abstract

Music performance anxiety (MPA) is a serious and prevalent problem among student musicians. Although previous studies have indicated the importance of social support from parents and teachers in the management of MPA, it remains unknown whether past social support influences pre-performance mental states and performance quality among student musicians. To address this, we asked 56 university-level music students to complete social support scales by recalling the social support they had received from their parents and a past music teacher before university enrollment, in addition to the social support they were receiving from a current music teacher. The students also recalled their most important public performance in the past six months, and they completed the Revised Competitive State Anxiety Inventory-2 and a performance evaluation scale. The results showed that social support from the past teacher positively predicted pre-performance self-confidence and performance quality. By contrast, social support from the current teacher positively predicted only performance quality and not pre-performance self-confidence. These findings highlight the differential roles of music teachers at different developmental stages of student musicians. Social support received from teachers by middle adolescence may be crucial for enhancing pre-performance self-confidence among student musicians.

## 1 Introduction

Music performance anxiety (MPA) is a form of anxiety triggered by public music performance situations (Salmon, 1990; Steptoe, 2001; Kenny, 2011; Gomez et al., 2023). MPA afflicts many musicians at different stages, including student musicians. Previous surveys indicated a high prevalence of MPA in this population (Fehm and Schmidt, 2006; Studer et al., 2011; Zakaria et al., 2013; Orejudo et al., 2018; Paliaukiene et al., 2018; de Lima et al., 2024). For example, 96% of music undergraduates reported experiencing MPA (Zakaria et al., 2013). Music students also reported higher MPA levels than professional musicians (Biasutti and Concina, 2014). MPA is a serious issue for student musicians, because it can negatively affect their music studies and careers (Fehm and Schmidt, 2006; Orejudo et al., 2018; Paliaukiene et al., 2018).

Previous literature suggests that social support from parents and teachers, which plays a crucial role in music learning (Gruber et al., 2008; Lehmann and Kristensen, 2014; Orejudo et al., 2021a), is also important in dealing with MPA. In several surveys, music students with MPA expressed the need for more support, especially from their teachers (Fehm and Schmidt, 2006; Studer et al., 2011; Ryan et al., 2021). An interview study also showed that student musicians relied on social support from parents and teachers to cope with MPA symptoms (Irie et al., 2023). Recent studies have indicated the effectiveness of social support in the management of MPA among student musicians. For example, Tahirbegi (2022) showed that positive teacher attitudes toward MPA management promoted help-seeking efforts among student musicians. Orejudo et al. (2021b) found that support from parents and teachers enhanced self-efficacy for learning, which further predicted self-efficacy for public performance, among students enrolled in advanced music schools. Kirsner et al. (2023) found that parental encouragement and support were associated with lower MPA levels. Moreover, Huang and Yu (2022) showed that social support from teachers helped student musicians improve their MPA coping skills.

These findings suggest the importance of social support from parents and teachers in MPA management. However, it remains unknown whether the social support received in the past affects pre-performance mental states and performance quality of student musicians. Orejudo et al. (2021b) recently found that social support from parents and teachers positively affected self-efficacy among student musicians aged 18 years or younger. However, the same study showed no evidence of the positive effects of social support from parents and teachers on self-efficacy among student musicians aged 19 years or older (Orejudo et al., 2021b). These results indicate that current social support can positively influence the mental states of student musicians at least until middle adolescence. Nevertheless, whether the positive effects of social support received by middle adolescence are retained after student musicians reach late adolescence or early adulthood remains underexplored. To address this, the present study aimed to examine whether and how past social support from parents and teachers affects pre-performance mental states and performance quality among university-level music students. More specifically, we examined whether the social support received from parents and teachers before university enrollment would influence students’ current levels of pre-performance anxiety, pre-performance self-confidence, and self-rated performance quality. Additionally, we examined whether social support from past and current teachers would have differential effects on the mental states and performance quality of music students.

## 2 Methods

### 2.1 Participants and procedure

Fifty-six undergraduate and postgraduate students majoring in music (50 women and six men; *M*_age_ ± *SD* = 21.5 ± 1.9 years; range: 18–28 years) at a university in Japan volunteered to participate in the present study. The proportion of men among the present participants (10.7%) was comparable to that among music students enrolled at this university, which ranged between 7.3 and 27.4% in the past ten academic years (2015–2024). According to a recent survey (Asaka, 2024), the proportion of men among graduates of music universities in Japan was 12.9% (academic years 1968–2020). Therefore, the gender ratio among the present participants was also comparable to that among music university students in Japan.

Of the 56 participants, 33 reported majoring in piano, 17 reported majoring in singing, four reported majoring in wind instruments, and two reported majoring in string instruments. On average, the participants started to play their major instrument or sing at the age of 8.8 ± 5.4 years. All the participants were classically trained musicians, but 32 reported playing other types of music (e.g., pop music and jazz) as well. The present study was approved by the National Institute of Advanced Industrial Science and Technology (AIST) Ethics Committee. All the participants provided written informed consent.

We asked the participants to recall the social support they had received from a past music teacher (i.e., a teacher who had given them lessons on their major instrument/singing) before entering university, and to complete the *teachers* subscale of the Social Support Scale developed by Ryan et al. (2000). Given that music students usually start to take lessons from a new teacher (e.g., professor) at the time of university enrollment in Japan (typically at the age of 18), the participants were also asked to complete the same questionnaire while recalling the social support they were receiving from their current teacher of their major instrument/singing. In addition, we asked the participants to recall the social support they had received from their parents before entering university, and to complete the *parents* subscale of the Social Support Scale (Ryan et al., 2000). Considering that a significant proportion of Japanese university students start to live separately from their parents at the time of university enrollment, we focused on parental support in the past (i.e., before entering university).

Additionally, the participants were asked to recall the most important public performance in the past six months. Of the 56 participants, 27 recalled concerts, 20 recalled exams, and nine recalled competitions. Given that MPA can sometimes lead to an actual impairment of performance quality (Yoshie et al., 2009a; Sokoli et al., 2022), the participants were asked to evaluate the quality of the recalled public performance. To assess the levels of anxiety and self-confidence experienced before the recalled public performance, we asked the participants to complete a modified version of the Revised Competitive State Anxiety Inventory-2 (CSAI-2R; Martens et al., 1990; Cox et al., 2003; Yoshie et al., 2009b).

### 2.2 Self-reported measures

#### 2.2.1 Social support

We assessed the level of perceived social support received from teachers by using a modified version of the *teachers* subscale of the Social Support Scale developed by Ryan et al. (2000). To investigate the influences of both past and current teachers, we asked the participants to recall two of their teachers: (1) a teacher who had given them lessons on their major instrument/singing before entering university (*past teacher*), (2) a teacher who was giving them lessons on their major instrument/singing (*current teacher*). If they had taken or were taking lessons from more than one teacher, they recalled the most influential teacher in the past and present. The participants were then asked to answer each of the original nine questions (e.g., “How much do/did you think your teacher is/was pleased with the work you do/did in music class?”) and nine additional questions (e.g., “How much do/did you think your teacher gives/gave you opportunities to rehearse in performance settings similar to actual public performances?”) on a scale ranging from 1 (*not very much*) to 7 (*a lot*), for each of the two teachers. Three experts from the domains of psychology, music pedagogy, and music performance discussed and devised the additional questions based on previous literature on MPA (e.g., Wolfe, 1990; Fehm and Schmidt, 2006; Osborne and Kenny, 2008; Klickstein, 2009).

To assess the level of perceived social support from parents, we asked the participants to complete the *parents* subscale of the Social Support Scale developed by Ryan et al. (2000). We asked the participants to recall the parental support they had received before entering university. The participants were then asked to answer each of the 12 questions (e.g., “How much did you think your parents would want you to pass music exams?”) on a scale ranging from 1 (*not very much*) to 7 (*a lot*). As the questionnaire survey was conducted in Japanese, we used a back-translation procedure to ensure translation validity.

#### 2.2.2 Pre-performance anxiety and self-confidence

Pre-performance anxiety and self-confidence were assessed by using a modified version of the CSAI-2R (Martens et al., 1990; Cox et al., 2003; Yoshie et al., 2009b). It includes three subscales: *cognitive anxiety* (five items; e.g., “I am concerned about choking under pressure.”), *somatic anxiety* (seven items; e.g., “My heart is racing.”), and *self-confidence* (five items; e.g., “I am confident I can meet the audience’s expectations.”). The CSAI-2 was originally developed to assess competitive state anxiety among athletes based on the multidimensional anxiety theory (Martens et al., 1990). Research in sport psychology has investigated the relationship between the three subscales of the CSAI-2 and athletic performance, and found that self-confidence positively and most significantly influences athletic performance (Craft et al., 2003; Woodman and Hardy, 2003). Several attempts have been made to apply the CSAI-2 or CSAI-2R to music performance situations (Miller and Chesky, 2004; Yoshie et al., 2008, 2009b; Yao and Li, 2022). Corroborating the findings of sport psychology research, self-confidence has been shown to positively and significantly affect performance quality among musicians (Yoshie et al., 2008, 2009b). By contrast, cognitive anxiety has been found to negatively affect the technical aspects of performance quality (Yoshie et al., 2009b).

In the present study, the participants were asked to recall the most important public performance in the past six months, and then to complete the modified version of the CSAI-2R. The participants were instructed to recall their feelings experienced during the two-week period prior to the specific public performance that they identified. For each item, the participants rated the symptom intensity level on a scale ranging from 1 (*not at all*) to 4 (*very much so*). The scores were averaged across items for each subscale. The mean score was multiplied by 10, leading to intensity scores ranging from 10 to 40 (Cox et al., 2003).

#### 2.2.3 Performance quality

The participants rated the quality of the recalled public performance by using a performance evaluation scale adapted from Yoshie et al. (2009a). The scale included ten items concerning both technical and artistic aspects (i.e., *accuracy*, *technical dexterity*, *tempo and rhythm*, *memory*, *artistry*, *interpretation*, *expressiveness*, *structural strength*, *melodic and harmonic balance*, and *tone quality*). The participants rated the quality of their public performance in relation to their normal level of performance quality (i.e., performance quality *during practice*). The scale ranged from 1 (*much worse*) to 9 (*much better*), with 5 indicating *the same level* as performance quality during practice. We employed this scale to capture intra-individual fluctuations in performance quality. We computed the mean scores for the ten items.

## 3 Results

Table 1 shows the means (SDs), internal consistency (Cronbach’s alpha coefficients), and inter-correlations among the CSAI-2R subscales, performance quality scale, and three social support scales. All the scales showed sufficient internal consistency, with Cronbach’s alpha coefficients ranging from 0.758 to 0.934.

**Table 1:** Descriptive statistics and intercorrelation matrix for the primary variables.

Variables	Mean	(SD)	α	1	2	3	4	5	6
1. Cognitive anxiety	21.23	(6.43)	0.758						
2. Somatic anxiety	21.10	(5.97)	0.778	0.678***					
3. Self-confidence	18.86	(5.87)	0.818	0.002	−0.010				
4. Performance quality	5.01	(1.27)	0.915	−0.269*	−0.289*	0.395**			
5. Past teacher	5.20	(0.82)	0.840	−0.079	−0.214	0.350**	0.410**		
6. Current teacher	5.18	(0.74)	0.837	−0.057	−0.047	0.290*	0.356**	0.313*	
7. Parents	5.17	(1.27)	0.934	−0.002	0.193	0.232	0.269*	0.270*	0.128

* *p* < 0.05, ** *p* < 0.01, *** *p* < 0.001.

First, we examined the relationship between the CSAI-2R subscales and performance quality. Pre-performance cognitive and somatic anxiety, measured using the CSAI-2R, were negatively correlated with performance quality (*r* = −0.269, *p* = 0.045; *r* = −0.289, *p* = 0.031). By contrast, self-confidence was positively correlated with performance quality (*r* = 0.395, *p* = 0.003). Therefore, lower levels of cognitive and somatic anxiety and higher levels of self-confidence during the two-week period before an important public performance were associated with better performance quality.

We then examined the relationship between the social support scales and the CSAI-2R subscales. Of the three CSAI-2R subscales, only self-confidence was significantly correlated with social support (Table 1). We found that social support from the past teacher was positively and significantly correlated with pre-performance self-confidence (*r* = 0.350, *p* = 0.009). Additionally, social support from the current teacher was positively and significantly correlated with pre-performance self-confidence (*r* = 0.290, *p* = 0.030). None of the social support scales showed a significant correlation with pre-performance cognitive or somatic anxiety. We subsequently performed a stepwise multiple regression analysis with the social support scales as the independent variables and the self-confidence subscale of the CSAI-2R as the dependent variable (Table 2). The social support scales accounted for a statistically significant portion of the self-confidence variance (*F* (1, 53) = 7.421, *p* = 0.009). We found that only social support from the past teacher significantly predicted pre-performance self-confidence (*β* = 0.350, *t* = 2.724, *p* = 0.009).

**Table 2:** Results of multiple regression analyses.

Variables	*β*	*t*	*p*
Self-confidence (*R^2^* = 0.123)			
Constant		1.147	0.256
Past teacher	0.350	2.724	0.009
Performance quality (*R^2^* = 0.231)			
Constant		0.008	0.994
Past teacher	0.327	2.554	0.014
Current teacher	0.265	2.071	0.043

Finally, we examined the relationship between the social support and performance quality scales. Performance quality during the important public performance relative to practice was positively and significantly correlated with all three social support scales (Table 1). Social support from the past teacher was most strongly correlated with performance quality (*r* = 0.410, *p* = 0.002). Social support from the current teacher was also significantly correlated with performance quality (*r* = 0.356, *p* = 0.007). In addition, social support from parents was weakly correlated with performance quality (*r* = 0.269, *p* = 0.045). A stepwise multiple regression analysis with the social support scales as the independent variables and the performance quality scale as the dependent variable (Table 2) demonstrated that the social support scales accounted for a statistically significant portion of the performance quality variance (*F* (2, 52) = 7.831, *p* = 0.001). We found that social support from both the past and current teachers significantly predicted self-rated performance quality (*β* = 0.327, *t* = 2.554, *p* = 0.014; *β* = 0.265, *t* = 2.071, *p* = 0.043).

## 4 Discussion

The present study examined how social support from parents and teachers could influence pre-performance anxiety, pre-performance self-confidence, and the quality of public performance among university-level music students. The finding that pre-performance self-confidence was positively associated with self-rated performance quality is consistent with previous studies demonstrating the positive influences of pre-performance self-confidence on both athletic performance (Craft et al., 2003; Woodman and Hardy, 2003) and music performance (Yoshie et al., 2008, 2009b). Although social support from past teachers positively predicted both pre-performance self-confidence and performance quality, social support from current teachers positively predicted only performance quality. We also found that social support from parents did not predict pre-performance self-confidence or performance quality.

These findings potentially highlight the differential roles of music teachers at different developmental stages of student musicians. The present participants typically entered university at the age of 18 years and were in late adolescence or early adulthood. Social support from teachers who had given students music lessons before university enrollment (i.e., by middle adolescence) enhanced both pre-performance self-confidence and the quality of public performance. By contrast, social support from current teachers who were giving students music lessons (i.e., during or after late adolescence) enhanced performance quality but did not affect pre-performance self-confidence. These results are consistent with a recent finding that social support from teachers does not significantly affect self-efficacy among university-level music students (Orejudo et al., 2021b). Interestingly, the same study identified an important role of teachers in enhancing self-efficacy among music students aged 18 years or younger (Orejudo et al., 2021b). Another recent study found that adolescent musicians (12–19 years old) with low levels of MPA perceived receiving more positive feedback from teachers than did those with high levels of MPA (Papageorgi, 2021). The developmental model of MPA also suggests the important role of teachers’ positive and supportive attitudes in preventing young musicians from developing MPA (Osborne and Kenny, 2008; Kenny, 2011; Patston and Osborne, 2016). Based on these previous studies, we postulate that social support from teachers positively influences the levels of pre-performance self-confidence among student musicians during or before middle adolescence and that this positive influence can remain even when they reach late adolescence or early adulthood.

The present findings imply that the roles of music teachers include not only teaching technical and musical requirements but also helping young musicians boost their self-confidence in public performances and manage MPA. This is consistent with the results of previous studies that have demonstrated the importance of teachers’ support in the management of MPA among student musicians (Orejudo et al., 2021b; Huang and Yu, 2022; Tahirbegi, 2022). More importantly, the present findings suggest that the positive influences of teachers’ support on students’ pre-performance mental states can persist even after students have left their teachers. It seemed that the social support received from teachers by middle adolescence was an important source of pre-performance self-confidence for student musicians. By contrast, the social support received from teachers during late adolescence or young adulthood did not have a significant impact on students’ pre-performance self-confidence. Based on these findings, we hypothesize the existence of a critical period for MPA interventions by music teachers. Therefore, we suggest that MPA management programs should be incorporated into music education for young learners during or before middle adolescence.

Unexpectedly, the present results failed to show any significant effects of past parental support. Perceived social support from parents was only weakly correlated with self-rated performance quality and did not predict pre-performance mental states or performance quality among student musicians. Nevertheless, accumulating evidence suggests the importance of parental support for young musicians (Orejudo et al., 2021b; Kirsner et al., 2023). Although the reasons for the minimal effects of past parental support in the present study remain unknown, one possible explanation is that the effects of past parental support on the mental states or performance quality of student musicians gradually weaken as the students grow older. Orejudo et al. (2021b) recently found that social support from parents significantly enhanced self-efficacy in student musicians aged 11–15 years. The positive effects of parental support were also found in student musicians aged 16–18 years, but peers also started to influence their self-efficacy. Interestingly, the positive effects of parental support on self-efficacy disappeared in student musicians aged 19 years or older (Orejudo et al., 2021b). These results suggest that parental support can positively influence the mental states of student musicians at least until middle adolescence. However, our results indicate that, unlike social support from teachers, the positive effects of past parental support may attenuate in late adolescence or early adulthood, when students expand relationships outside of their families.

The present study has several limitations. First, we recruited a relatively small sample of Japanese musicians. Reflecting the strong gender gap among Japanese music students, the male-to-female ratio of the sample was unbalanced. Future research should attempt to recruit a larger sample with a balanced gender ratio to examine potential gender differences in the perception of social support. Given that Japanese culture might have influenced the present results, future research should also examine whether these results would be replicated in other cultures. Second, the present findings were based on retrospective self-ratings of social support received in the past. Thus, the participants’ responses could be influenced by memory distortion. Future studies should adopt longitudinal approaches to address this issue. Finally, the present study focused only on the influences of parents and teachers. Since recent studies have also indicated the importance of social support from peers (Orejudo et al., 2021b; Huang and Yu, 2022), future research should examine how past peer support or peer learning can affect pre-performance mental states and performance quality among student musicians.

In summary, the present study suggests that the social support received from teachers by middle adolescence plays an important role in helping student musicians manage MPA. Social support from past teachers enhances pre-performance self-confidence and the quality of public performance among university-level music students. The positive influences of teachers can persist even after students leave them and reach late adolescence or young adulthood. These findings highlight the importance of early intervention for MPA by music teachers.

## Data Availability

The deidentified questionnaire data supporting the conclusions of this article will be made available within the scope of consent of the participants. Further inquiries can be directed to the corresponding author.
